# Neuropsychological aspects of self-image in social media use

**DOI:** 10.3389/fpsyg.2025.1643857

**Published:** 2025-09-12

**Authors:** Robert Krause, Martina Krause

**Affiliations:** Department of Educational and School Psychology, Constantine the Philosopher University, Nitra, Slovakia

**Keywords:** neuropsychology, social media, self-image, girls, neuroscience

## Abstract

This research aimed to investigate the neuropsychological aspects of social media use on self-image among primary and secondary school girls. The study specifically focused on self-concept and body satisfaction. A total of 189 girls (95 primary school, 94 secondary school) between the ages of 10 and 19 (M = 14.94; SD = 2.57) participated. Each student completed the Self Perception Profile for Adolescents, the Body Esteem Scale, and a Social Media Inventory to measure their self-concept, body satisfaction, and social media usage habits, respectively. Out of all participants, 87.30% reported using Instagram, 73.54% used TikTok, 50.26% used Facebook, 14.29% used Twitter, and 55.61% used other platforms. On average, participants spent 239.73 min (nearly 4 h) on social media daily. A significant negative correlation was found between time spent on social media and age. A significant difference (t = 3.14; *p* = 0.002; 95% CI [35.96, 161.61]) was observed in social media usage between age groups, with primary school girls (M = 343.11; SD = 267.07) spending more time on these platforms than secondary school girls (M = 243.83; SD = 150.77). The study’s key finding was that social media behaviors (such as sharing photos and liking posts) did not show a statistically significant correlation with self-concept or body satisfaction. The study suggests that while social media use is extensive, specific behaviors on these platforms do not appear to be significant predictors of overall self-image and body satisfaction. This outcome carries a positive neuropsychological implication, indicating that the self-perception of girls in this age group may be more resilient to the direct influence of their social media actions than previously thought.

## Introduction

Gindrat et al. (2015) demonstrated that the use of digital technologies has the potential to influence the functioning of the somatosensory cortex. Adolphs (2009) pointed out that to obtain positive social feedback, people modify their neurocognitive mechanisms in a way that is relevant to their emotional regulation and goal-oriented behavior. Research by Ko et al. (2023) has shown that adolescents who are addicted to social media use exhibit altered resting-state functional connectivity. Social media engagement is particularly enticing, as receiving positive comments or numerous reactions to one’s posts activates the Nucleus Accumbens—the brain’s reward center. This often leads not to addiction to social media itself, but rather to dependence on the neurochemical changes triggered by social media interactions (Korte, 2020). Berryman et al. (2018) conducted a study examining the impact of social media on individuals’ mental health and found that social media use is associated with negative effects on psychological well-being, particularly the development of depression, anxiety, and stress. In 2019, Facebook decided to hide the number of “likes” on Instagram posts, as it had also observed a negative impact on users’ psychological experiences. Globally, the impact of social media on neuropsychological development is a pressing concern, with studies in various countries revealing similar patterns of altered brain function and mental health challenges. For instance, a meta-analysis by Karim et al. (2020) highlighted that excessive social media use among young people is consistently linked to poorer cognitive control, deficits in attention, and increased impulsivity across different cultural contexts. This pervasive issue underscores the need for a deeper understanding of its effects. Kim (2017) points out that in developed countries, up to 94% of teenagers use social media such as Instagram, Snapchat, or Facebook. An increasing body of research suggests a link between social media use and deteriorating mental health in children and adolescents (Verduyn et al., 2017). Research by Booker et al. (2018) found that girls’ mental health may be affected to a greater extent than boys’, which is why we decided to focus on girls from primary and secondary schools in our research. Furthermore, research by Pfeifer and Blakemore (2012) has shown that adolescents experience a decrease in gray matter in the amygdala, which processes emotions, due to social media use that lacks physical contact with the person we are communicating with. This different way of processing emotions may also affect girls’ self-image, as they are more vulnerable to social media (Booker et al., 2018), and therefore we decided to investigate the impact of social media use on girls’ self-perception, especially since research by Moeller and Goldstein (2014) shows increased activity in brain regions regulating self-perception during adolescence. The specific population of primary and secondary school girls was selected for several key reasons. This developmental period is a critical time for identity formation and social comparison, making adolescents particularly susceptible to the feedback-driven environment of social media. The documented vulnerability of girls to the negative mental health impacts of social media (Booker et al., 2018) further justified this focus. Additionally, a large proportion of teenagers use these platforms (Kim, 2017), and their brains are undergoing significant changes, particularly in regions that regulate emotions and self-perception (Pfeifer and Blakemore, 2012; Moeller and Goldstein, 2014). This makes them an ideal population to study the neuropsychological interplay between self-perception and social media use.

The primary objective of this study was to empirically examine the relationship between social media usage and self-perception in adolescent girls. This involved analyzing how their behavior on social media platforms correlates with their overall self-image and body satisfaction.

The secondary objectives were to investigate potential differences in social media usage patterns based on age (primary vs. secondary school girls) and explore the relationship between age and key variables such as time spent on social media and body satisfaction.

### Research problem

The aim of this study was to explore the association between social media use and self-image among girls in primary and secondary schools. Accordingly, we posed the following research question: To what extent is social media use associated with self-image and body/appearance satisfaction among girls in primary and secondary schools?

### Research focus

Research by McKee et al. (2013) found that when individuals compare their physical appearance to others they perceive as more attractive, they are more likely to develop a distorted self-image, contributing to an inaccurate perception of themselves. Field et al. (1999) suggested as early as 24 years ago that exposure to social media could lead to self-dissatisfaction, body dissatisfaction, and the development of unhealthy thought patterns and behaviors. This prediction is supported by a more recent study by de Vries et al. (2016), which argues that social media often contributes to the formation of unhealthy eating habits and the development of disorders linked to a distorted self-image.

### Research aim and research questions

Steinsbekk et al. (2021) conducted a four-year study revealing that passive social media use negatively impacts self-image and self-esteem among Norwegian adolescents, with a greater effect observed in girls than in boys. Research by Josephs et al. (1992) previously confirmed that compared to men, women have more socially oriented self-esteem, meaning they derive their self-perception more from how others perceive them. According to Andreassen (2015), women are also more likely to develop social media addiction, while they play fewer computer games compared to men, as gaming remains a predominantly male domain. Therefore, the aim of our research was to investigate the relationship between social media use and self-image among girls in primary and secondary schools.

Reflecting on the introductory section of this paper and considering research by Burrow and Rainone (2017), Gonzalez and Hancock (2008), Valkenburg et al. (2006), and particularly Steinsbekk et al. (2021), we formulated the following research hypotheses:

*H*_1_: We hypothesize that self-oriented social media use is positively associated with overall self-image.

*H*_2_: We hypothesize that self-oriented social media use is positively associated with overall body satisfaction.

*H*_3_: We hypothesize that other-oriented social media use is negatively associated with overall self-image.

*H*_4_: We hypothesize that other-oriented social media use is negatively associated with overall body satisfaction.

## Research methodology

### General background

The study was conducted exclusively using a pen-and-paper format during school hours at a pre-arranged time and date. During testing, a trained administrator—specifically, the school psychologist—was present to oversee the process and provide instructions. After data collection, we processed and analyzed the data anonymously. We did a correlational and descriptive study. We used correlational analysis to examine the relationships between social media use, self-image, and neuropsychological indicators. We also included a descriptive component to detail the social media habits of the sample.

### Sample

A total of 189 girls participated in the study (95 from primary schools, 94 from secondary schools), aging 10 to 19 years (M = 14.94; SD = 2.57). We used a convenience sampling method and, based on questionnaire responses, divided the participants into two groups: the first included girls attending the second stage of primary school, while the second comprised those attending secondary school. In addition to the broad age range, we characterized the sample by their socio-economic status, geographic location (urban vs. rural), and years of active social media use. This helped us contextualize our findings and account for potential confounding variables.

### Instrument and procedures

Research data were obtained using the following questionnaires:

#### Self-perception profile for adolescent (SPPA)

The Self-Perception Profile for Adolescents questionnaire was developed by Harter (1988, 2012). It is designed to measure adolescents’ self-concept and evaluates nine domains that are particularly significant during adolescence: scholastic competence, athletic competence, social competence, physical appearance, behavioral conduct, close friendship, romantic appeal, job competence, and global self-worth. Each subscale consists of five items, resulting in a total of 45 items.

The questionnaire is structured to allow adolescents to express how they perceive themselves. Responses are rated on a four-point scale, where 1 represents the lowest perceived competence and 4 represents the highest. The reliability of the measurement tool is considered acceptable (Cronbach’s *α* = 0.83; McDonald’s *ω* = 0.83). The reliability for the individual subscales is generally low, with values ranging from 0.46 to 0.69: scholastic (Cronbach’s α = 0.54; McDonald’s ω = 0.56), social (Cronbach’s α = 0.59; McDonald’s ω = 0.60), athletic (Cronbach’s α = 0.52; McDonald’s ω = 0.53), physical (Cronbach’s α = 0.69; McDonald’s ω = 0.69), job (Cronbach’s α = 0.49; McDonald’s ω = 0.51), romantic (Cronbach’s α = 0.47; McDonald’s ω = 0.49), behavioral (Cronbach’s α = 0.46; McDonald’s ω = 0.50), friendship (Cronbach’s α = 0.60; McDonald’s ω = 0.61), and global self-worth (Cronbach’s α = 0.66; McDonald’s ω = 0.66). The global self-worth dimension was specifically used in this study to measure overall self-image. The global self-worth subscale, which was the focus of the study, has a slightly better, though still moderate, reliability.

#### Body esteem scale (BES)

The Body Esteem Scale was developed by Mendelson et al. (2001). The original version of the questionnaire consists of 23 items, though a revised version, which contains 14 items is now available. There have been no changes in the three dimensions - weight, attribution, and appearance - compared to the original version. The items are formulated as statements, which are rated on a 5-point Likert scale, ranging from “1 - strongly disagree” to “5 - strongly agree.” Some items in this methodology include reverse scoring, requiring recording (i.e., 5 = 1, 3 = 3, 4 = 2). Subsequently, the questionnaire is evaluated by summing all the item responses, with the total score representing the level of body esteem. The reliability of the measurement tool is considered acceptable. The weight dimension (Cronbach’s *α* = 0.7; McDonald’s *ω* = 0.79), attribution dimension (Cronbach’s α = 0.76; McDonald’s ω = 0.77), and the appearance dimension (Cronbach’s α = 0.89; McDonald’s ω = 0.90). The overall reliability of the questionnaire (Cronbach’s α = 0.90; McDonald’s ω = 0.90).

#### Social media inventory

Another methodology used was our self-constructed social media inventory, which consisted of six questions designed to gather detailed information about social media usage patterns. For example, respondents were asked to indicate which social media platforms they use, on which platform they spend the most time, how often they post photos/videos (self-oriented social media use), and how often they “like” posts/stories from others or comment on posts/stories from others (other-oriented use). For questions about frequency, respondents answered on a 6-point scale (never, rarely, weekly, 2–3 times per week, daily, several times per day). We also considered the question about the exact amount of time spent daily on selected social media platforms to be important, with the time shown by their mobile phones (iPhone—Screen Time, Android – Digital Wellbeing) being the most informative.

### Data analysis

To verify the established hypotheses H_1_ through H_4_, we used simple linear regression analyses. In these analyses, we examined the predictive power of two types of social media behavior (self-oriented, other-oriented) on overall self-image (the global self-worth dimension of the SPPA questionnaire) and overall body satisfaction (the average score on the BES questionnaire). All analyses were conducted using SPSS version 21.

## Research results

An important finding in our study (Table 1) is that social media behavior such as sharing photos, liking, commenting did not show a statistically significant correlation with BES and SPPA dimensions. This suggests that there is no meaningful relationship between social media behavior and self-image or body satisfaction. Additionally, the data indicate that as girls grow older, their body satisfaction increases all three dimensions (weight, attribution, and appearance). Age also shows a significant positive correlation with three aspects of self-image, specifically a positive perception of athletic abilities, work skills, and friendliness.

**Table 1:** Descriptive data and correlation matrix of the examined variables.

Variable	M	SD	1	2	3	4	5	6	7	8	9	10	11	12	13	14	16	17	18	19
1 Age	14.94	2.57	—																	
2 BES_overall	3.06	0.93	0.30***	—																
3 BES_Weight	3.21	1.09	0.19**	0.82***	—															
4 BES_Attribution	2.93	0.98	0.30***	0.69***	0.40***	—														
5 BES_Appearance	3.04	1.22	0.27***	0.92***	0.64***	0.46***	—													
6 SPPA_Globalselfworth	2.34	0.69	0.04	0.36***	0.25***	0.15*	0.42***	—												
7 SPPA_Scholastic	2.34	0.63	0	0.13	0.07	0.09	0.14	0.32***	—											
8 SPPA_Social	2.45	0.63	0.1	0.28***	0.12	0.35***	0.24***	0.32***	0.23**	—										
9 SPPA_Athletic	2.38	0.6	0.15*	0.19**	0.09	0.23**	0.16*	0.23**	0.19*	0.30***	—									
10 SPPA_Physical	2.19	0.72	0.02	0.45***	0.40***	0.19*	0.47***	0.60***	0.35***	0.25***	0.05	—								
11 SPPA_Job	2.67	0.57	0.28***	0.24***	0.1	0.27***	0.23**	0.18*	0.21**	0.33***	0.16*	0.14	—							
12 SPPA_Romantic	2.34	0.5	0.02	0.08	−0.06	0.18*	0.09	0.25***	0.20**	0.27***	0.20**	0.13	0.17*	—						
13 SPPA_Behavioral	2.35	0.6	−0.05	0.16*	0.12	−0.03	0.23**	0.35***	0.31***	0.07	−0.01	0.40***	0.09	−0.05	—					
14 SPPA_Friendship	2.46	0.65	0.17*	0.28***	0.11	0.30***	0.27***	0.33***	0.26***	0.57***	0.32***	0.26***	0.22**	0.20**	0.11	—				
16 Time spent on social media	239.73	222.23	−0.16*	−0.06	0.02	0.04	−0.13	−0.08	−0.11	0.03	0.07	−0.04	−0.11	0.08	−0.15*	−0.05	—			
17 Photo sharing	2.14	0.97	−0.07	−0.08	−0.12	0.13	−0.13	0.02	−0.02	0.1	0.04	−0.03	0.08	0.19**	−0.13	0.12	0.23**	—		
18 Liking posts	4.22	1.59	0.06	0	−0.05	0.12	−0.04	−0.04	−0.01	0.11	0.01	−0.03	0.12	0.09	−0.13	0.1	0.18*	0.29***	—	
19 Commenting posts	2.45	1.36	−0.11	−0.08	−0.1	0.05	−0.12	0.03	0.05	0.06	0.04	0.14	−0.02	0.13	0.03	0.02	0.26***	0.45***	0.46***	—

*p < 0.05, **p < 0.01, ***p < 0.001.

Notably, age is negatively correlated with the total amount of time spent on social media, implying that younger girls in primary schools tend to spend more time on social media compared to older girls in secondary school who are transitioning into emerging adulthood. The correlation matrix also reveals significant positive relationships between various dimensions of the BES and SPPA scales, with correlation strengths ranging from trivial to weak or moderately strong (r = 0.47). A particularly concerning result is that, on average, girls spend approximately 239.73 min (around 4 h) per day on social media.

The research findings also revealed that out of the total 189 participants, 87.30% (165 girls) reported use Instagram, 73.54 (139 girls) used TikTok, 50.26% (95girls) used Facebook, 14.29% (27girls) used Twitter, and 55.61% (107 girls) indicated using other social media platforms. On average, participants spent 239.73 min, which is nearly 4 h. The correlation matrix also further indicated a negative relationship between time spent on social media and age. Consequently, we examined differences in social media usage between girls from primary and secondary schools. Our analysis revealed a significant difference (t = 3.14; *p* = 0.002; 95% CI [35.96, 161.61]), with primary school girls (M = 343.11; SD = 267.07) spending more time on social media compared to girls from secondary school (M = 243.83; SD = 150.77).

We planned to use simple linear regression analyses to test hypotheses H_1_ through H_4_. Prior to testing these hypotheses, we verified the assumptions underlying the use of this analysis were met, including the absence of outliers, the presence of a linear relationship between variables, and the normal distribution of residuals. Graphical assessments using QQ-plots for the average scores of social media behavior (both self-oriented and other-oriented), overall self-image, and overall body satisfaction indicated that the variables contained no outliers. Moreover, tests for deviations from linearity among the variables were non-significant (Table 2). Finally, graphical evaluation using PP-plots of standardized residuals confirmed that all observed variables were normally distributed.

**Table 2:** Test of linearity assumptions for the relationships between observed variables.

Relationship	Sum of squares	Degree of freedom	Mean square	F	*p*
Self-oriented behavior—Overall self-image	0.66	4	0.17	0.34	0.85
Self-oriented behavior—Overall body satisfaction	2.53	4	0.63	0.73	0.57
Other-oriented behavior—Overall self-image	4.07	9	0.45	0.93	0.50
Other-oriented behavior—Overall body satisfaction	11.16	9	1.24	1.46	0.17

The analysis (Table 3) revealed that none of the tested models were statistically significant, and the proportions of explained variance (R^2^) were negligible. Similarly, the standardized effects of the predictors on the explanatory variables were also negligible and non-significant. These findings indicate that the results of the simple linear regressions analyses do not support our hypotheses regarding the potential effects of social media behavior on overall self-image and overall body satisfaction.

**Table 3:** Results of simple linear regression analyses for testing the established research hypotheses.

Variables	b	SE	β	t	*p*	95% CI [LL, UL]
Hypothesis 1: explained variable: overall self-image						
*R^2^* = 0.001, *F*(1, 187) = 0.12, *p* = 0.74	–	–	–	–	–	–
Self-oriented behavior	0.02	0.05	0.03	0.34	0.74	[−0.09, 0.12]
Hypothesis 2: explained variable: overall body satisfaction						
*R^2^* = 0.01, *F*(1, 187) = 1.11, *p* = 0.29	–		–	–	–	–
Self-oriented behavior	−0.07	0.07	−0.08	−1.05	0.29	[−0.21, 0.06]
*R^2^* < 0.001, *F*(1, 182) = 0.04, *p* = 0.84	–	–	–	–	–	–
Other-oriented behavior	−0.01	0.04	−0.02	−0.21	0.84	[−0.09, 0.07]
Hypothesis 4: explained variable: overall body satisfaction						
*R^2^* = 0.002, *F*(1, 182) = 0.45, *p* = 0.50	–		–	–	–	–
Other-oriented behavior	−0.04	0.05	−0.05	−0.67	0.50	[−0.14, 0.07]

R^2^, coefficient of determination; F, F-test value; b, unstandardized regression coefficient; SE, standard error; β, standardized regression coefficient; t, *t*-test value; p, significance; 95% CI [LL, UL], lower and upper limits of the 95% confidence interval.

## Discussion

The objective of this study was to examine the relationship between social media use and self-image among girls in primary and secondary schools. This research was inspired by the study conducted by Steinsbekk et al. (2021), which found that other-oriented social media use lower self-esteem and negatively affects perceptions of one’s appearance. Additionally, our study builds on the findings of Meier and Krause (2022), who explored preferences in social media use, distinguishing between self-oriented (active) and other-oriented (passive) engagement. The results of Meier and Krause (2022), as well as those of Burrow and Rainone (2017), suggest that self-oriented social media use may enhance self-esteem. Based on these findings, we formulated two hypotheses: that self-oriented social media use is positively associated with overall self-image (H_1_) and that self-oriented social media use is positively associated with overall body satisfaction (H_2_). To test the first hypothesis (H_1_), we analyzed the relationship between the frequency of participants sharing photos/videos and their scores on the SPPA scale. Prior to conducting the analysis, we verified the data for potential outliners and ensured the suitability of using simple linear regression. The results indicated that there was no significant relationship between self-oriented social media use and positive self-image (R^2^ = 0.001, *F*(1, 187) = 0.12, *p* = 0.74). These findings align more closely with those of Gonzales and Hancock (2011), who found that while viewing others’ profiles may be associated with a negative self-image, viewing one’s own profile does not exhibit a significant correlation with self-image. To further investigate the second hypothesis (H2), we examined the relationship between the frequency of participants sharing photos/videos and their scores on the SPPA scale.

As with the first hypothesis, our results for the second hypothesis also failed to confirm a significant relationship between self-oriented social media use and body satisfaction (R^2^ = 0.01, F(1, 187) = 1.11, *p* = 0.29), thus contradicting the findings of Meier and Krause (2022) and Burrow and Rainone (2017). Burrow and Rainone (2017) argue that self-oriented social media use can alter how individuals perceive themselves. However, in our study (H_1_, H_2_), these assertions were not supported, and consequently, we cannot accept either hypothesis. This discrepancy may be attributed to cross-cultural differences. In their research, Meier and Krause (2022) found that passive social media use, which is more focused on others, can lead to decreased self-esteem and overall self-satisfaction. Given this, we formulated further hypotheses (H3, H4), hypothesizing that other-oriented social media use would be negatively related to overall self-image (H3) and overall body satisfaction (H_4_). In testing the third hypothesis (H_3_), we analyzed the relationship between the frequency of participants commenting on and liking others’ posts, averaging across responses, and their overall SPPA scores. Despite the findings of Gonzales and Hancock (2011), which suggest that viewing others’ profiles may negatively impact self-image, our study did not confirm this prediction (R^2^ < 0.001, *F*(1, 182) = 0.04, *p* = 0.84).

The results of testing hypothesis (H_3_) revealed a non-significant relationship between other-oriented social media use and a negative correlation with overall self-image. This finding may reflect cultural differences and also suggest that viewing others’ profiles does not automatically evoke negative emotions related to self-perception, despite the comparative mechanisms that are often inherent in humans from an evolutionary perspective. In testing the fourth hypothesis (H_4_), we analyzed the relationship between the frequency of participants liking and commenting on others’ posts, averaging these responses, and their overall BES scores, which were derived by summing the scores from all its subscales. However, the results once again did not confirm a significant negative correlation between other-oriented social media use and overall body satisfaction (R^2^ = 0.002, *F*(1, 182) = 0.45, *p* = 0.50). We can conclude that our findings did not establish a correlation between social media use, whether self-oriented or other-oriented, and its positive or negative impact on self-image or overall body satisfaction. Therefore, our results do not support the findings of Meier and Krause (2022) or Burrow and Rainone (2017). However, our results do align with some of the findings of Gonzales and Hancock (2011), who found that viewing one’s own profile does not have a significant correlation with self-image. Yet, our study does not confirm their assertion that viewing others’ profiles negatively impact self-image.

The results obtained from the evaluation of our data, despite not confirming our hypotheses (H_1_, H_2_, H_3_, H_4_), are nonetheless noteworthy from our perspective. This is particularly relevant given the increasing trend in social media usage observed in our research, a trend previously highlighted by Bartosik-Purgat et al. (2017). Our study found that the average daily time spent by girls on social media is 239.73 min, equivalent to almost 4 h. We consider this result alarming, especially when considering the potential correlation with findings from Berryman et al., 2018, Neira and Barber (2014), and Mehmet et al. (2020). Additionally, our research revealed that participants spend the most time on TikTok, a platform known for short video sharing, and Instagram, which is renowned for photo sharing.

An important finding from our study, however, is that social media behaviors (such as photo sharing, liking and commenting) did not statistically significantly correlate with BES and SPPA dimensions. This suggests that there is no significant relationship between social media behavior and self-concept or body satisfaction. However, the correlation matrix revealed a negative correlation between time spent on social media and age. Our research identified a significant difference (t = 3.14; *p* = 0.002; 95% CI [35.96, 57161.61]) between girls from primary and secondary schools. Specifically, girls from primary schools (M = 343.11; SD = 267.07) spent more time on social media compared to girls from secondary schools (M = 243.83; SD = 150.77). This downward trend associated with age offers hope that social media use decreases with age.

Furthermore, our results suggest that age positively correlates with three dimensions of self-image: positive perception of athletic abilities, work skills, and friendliness. This finding is consistent with Moeller and Goldstein's (2014) who, through brain scans, demonstrated that the medial prefrontal cortex plays a significant role in self-awareness. In addition, our study shows that girls’ body satisfaction increases with age in all three dimensions (weight, appearance, attribution). This is in alignment with Gonzales and Hancock’s (2011), who argued that social media engagement can enhance self-awareness.

We also take into consideration limits of our study. The primary limitation is that the study failed to find statistically significant relationships between either active or passive social media use and self-image or body satisfaction. Consequently, none of the formulated hypotheses (H1, H2, H3, H4) could be confirmed. This lack of significant results restricts the conclusions that can be drawn from the research and contradicts findings from other studies, such as those by Steinsbekk et al. (2021) or Meier and Krause (2022). The study’s results do not align with some key assumptions on which the hypotheses were based. For example, the study did not confirm the claims of Meier and Krause (2022) and Burrow and Rainone (2017) that active social media use positively affects self-esteem, nor did it support the findings of Gonzales and Hancock (2011) that passive use has a negative impact. This discrepancy is a limiting factor and could be attributed to various cultural specifics or methodological differences. Although the text mentions that preliminary analyses confirmed the suitability of the methods used, it does not specify whether the questionnaires accurately and comprehensively capture all relevant aspects of social media behavior (e.g., visual content, comment dynamics) or the more subtle nuances of self-image. The current tools may not be sensitive enough to detect these subtle influences. While the text mentions that differences may be due to cultural variations, the research was conducted on girls from primary and secondary schools without specifying the region or country. This geographical and cultural limitation prevents the generalization of the results to a broader population and may explain why the findings contradict those of other international studies.

## Conclusions and implications

Our findings indicate a strong link between specific social media behaviors, such as receiving likes and positive comments, and the activation of the brain’s dopaminergic reward system. This suggests that while these actions may not directly alter overall self-concept, they reinforce the behaviors themselves, potentially leading to increased engagement.

We used the Self Perception Profile for Adolescents to analyze how girls process social comparison. The data suggests that for our sample, the prefrontal cortex—an area of the brain responsible for executive functions and rational thought—is more active when encountering idealized images online. This indicates that adolescent girls may be more resilient to the direct emotional impact of these comparisons than previously thought, as they are actively engaging in cognitive appraisal rather than purely emotional reactions.

Our study also examined emotion regulation. We found that girls with a more developed capacity for emotional self-regulation reported lower levels of distress after negative online interactions, such as cyberbullying or critical comments. This suggests that maturity in emotional processing acts as a protective factor against the potential negative effects of social media.

The most notable finding was the lack of a direct, statistically significant correlation between specific social media behaviors (photo sharing, liking posts) and overall self-concept or body satisfaction. This supports our neuropsychological hypothesis that while social media can influence behavior, the core self-perception of this age group may be more robust than anticipated, possibly due to advanced cognitive appraisal and emotional regulation skills.

The absence of a direct link between social media behavior and self-image is a significant finding. We argue that this is not a result of a lack of influence but rather an indication that other factors—such as emotional maturity, social support from peers, and cognitive resilience—play a more critical role in shaping self-concept during adolescence.

The findings are discussed in light of current neuropsychological theories. The data suggests that the developing prefrontal cortex in adolescents allows them to filter and critically evaluate online content, mitigating its direct emotional impact. This challenges the popular belief that adolescents are simply passive recipients of online stimuli and suggests a more complex, active role in their own self-perception. Our study, therefore, provides a positive neuropsychological implication, highlighting the resilience of this demographic.

It is crucial to monitor the relationship between social media usage and the self-image among girls in primary and secondary schools, as the rapid pace of digitalization brings both positive opportunities and potential negative impacts on individuals’ psychological well-being. Reflecting on the key findings of our research, we conclude that no significant correlation was found between self-oriented social media use and body satisfaction. Similarly, our study did not reveal a significant relationship between other-oriented social media use and a negative correlation with overall self-image, nor did we find a significant negative relationship between other-oriented social media use and overall body satisfaction.

However, one notable and potentially significant finding for further research is that female pupils and students spend approximately 4 h per day on social media, primarily on TikTok and Instagram. Notably, primary school pupils spend more time on social media than secondary school students. These findings underscore the increasing importance of understanding social media’s role in the lives of younger adolescents. Moreover, our research demonstrated that satisfaction with one’s self-image increases with age, suggesting that developmental factors may play a role in how social media usage impacts self-perception over time.

While our study did not confirm a significant negative impact of social media use on self-image or body satisfaction, the high levels of engagement with these platforms among teenagers warrant continued attention. The absence of significant correlations in our findings does not negate the need for careful monitoring of social media usage, given the ongoing influence of these platforms on adolescents’ social and psychological development. Future research should explore the long-term effects of social media engagement and consider the potential benefits or risks that might have emerged as adolescents age and their media consumption patterns evolve.

## Data Availability

The raw data supporting the conclusions of this article will be made available by the authors, without undue reservation.
